# Long-lived charge carrier generation in ordered films of a covalent perylenediimide–diketopyrrolopyrrole–perylenediimide molecule†
†Electronic supplementary information (ESI) available: Experimental details including synthetic methods, electrochemical characterization, and spectroscopic characterization of molecules **2** and **3** are given as described in the text. See DOI: 10.1039/c4sc02551b



**DOI:** 10.1039/c4sc02551b

**Published:** 2014-09-16

**Authors:** Patrick E. Hartnett, Scott M. Dyar, Eric A. Margulies, Leah E. Shoer, Andrew W. Cook, Samuel W. Eaton, Tobin J. Marks, Michael R. Wasielewski

**Affiliations:** a Department of Chemistry and Argonne-Northwestern Solar Energy Research (ANSER) Center , Northwestern University , Evanston , Illinois 60208-3113 , USA . Email: m-wasielewski@northwestern.edu ; Email: t-marks@northwestern.edu ; Fax: +1-847-467-1425 ; Tel: +1-847-467-1423

## Abstract

The photophysics of a covalently linked perylenediimide–diketopyrrolopyrrole–perylenediimide acceptor–donor–acceptor molecule (PDI–DPP–PDI, **1**) were investigated and found to be markedly different in solution *versus* in unannealed and solvent annealed films. Photoexcitation of **1** in toluene results in quantitative charge separation in *τ* = 3.1 ± 0.2 ps, with charge recombination in *τ* = 340 ± 10 ps, while in unannealed/disordered films of **1**, charge separation occurs in *τ* < 250 fs, while charge recombination displays a multiexponential decay in ∼6 ns. The absence of long-lived, charge separation in the disordered film suggests that few free charge carriers are generated. In contrast, upon CH_2_Cl_2_ vapor annealing films of **1**, grazing-incidence X-ray scattering shows that the molecules form a more ordered structure. Photoexcitation of the ordered films results in initial formation of a spin-correlated radical ion pair (electron–hole pair) as indicated by magnetic field effects on the formation of free charge carriers which live for ∼4 μs. This result has significant implications for the design of organic solar cells based on covalent donor–acceptor systems and shows that long-lived, charge-separated states can be achieved by controlling intramolecular charge separation dynamics in well-ordered systems.

## Abstract 2

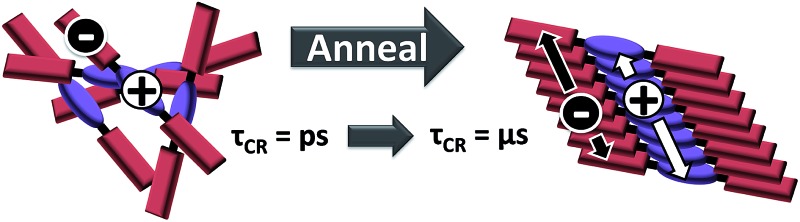
Self-ordering of covalent electron donor–acceptor building blocks in thin films upon solvent vapor annealing results in a 10^4^ increase in photo-generated charge carrier lifetime.

## Introduction

Charge transfer in organic molecules has been an area of intense research for decades and is a particularly important process in the field of organic electronics. Recently, with the rapidly increasing performance of organic photovoltaics (OPVs), the physics underlying charge transfer between the donor and acceptor in the active layer has been widely investigated with the goal of obtaining insights which could afford even higher PV performance.^1–5^ Currently, the highest-performing systems are based on interpenetrating blends of either a polymer or small molecule donor and a fullerene-based acceptor, to form a bulk-heterojunction (BHJ) active layer.^6,7^ Since BHJs are inherently disordered, defining general mechanisms describing, and ultimately design rules based on, charge separation in these systems presents a major challenge.^8,9^ Furthermore, the wide variety of donors and morphologies involved in BHJ OPVs adds complexity to unravelling what may be completely different mechanisms of charge separation.^10^

In contrast, covalent molecular donor–acceptor dyad or triad systems in solution are generally monodisperse and consequently the photophysics of such systems are much better understood and can be more easily manipulated. In covalent systems, the rates of charge separation and recombination can usually be described reasonably well by Marcus theory.^11^ These rates can be tuned in a predictable fashion by changing the redox potentials of the donor and/or acceptor, the electronic coupling between the donor and acceptor, and the dielectric constant of the solvent.^12,13^ Interestingly, using the same model to explain the photophysics observed in high performance BHJs is not straightforward.^5,14,15^ In particular, the electron transfer rate is often significantly faster than expected, and the high yield of free charge carriers observed in BHJs contradicts what is predicted by Onsager–Braun ion pair interaction theory.^4,16^ This theory predicts that under typical OPV operating conditions, the majority of ion pairs are coulombically bound in a radical ion pair (RP) state, but in practice, internal quantum efficiencies approaching 100% have been observed.^17,18^ This result has been explained by excess energy in the RP state, charge carrier delocalization, and entropic effects.^1,19–22^

Although OPVs have recently surpassed 10% power conversion efficiency (PCE), reliance on fullerene acceptors remains a major barrier to widespread adoption of this technology. Fullerenes are expensive, unstable towards oxidation in air, and are not easily modified to absorb light. Recent research has seen OPVs based on non-fullerene small molecule acceptors achieve efficiencies over 4%, and all-polymer cells have been reported with an exceptional efficiency of 6.4%. Nevertheless, significant research will be required to find suitable fullerene replacements.^23–26^ Some of the highest efficiency non-fullerene acceptors are based on naphthalenediimide (NDI) and perylenediimide (PDI) derivatives.^25,27–29^ These materials are inexpensive to synthesize, easily modified, and chemically/environmentally robust. However, unlike fullerenes, which have π-systems that can transfer charge in three dimensions, NDIs and PDIs are essentially one-dimensional conductors as a result of their pronounced tendency to π-stack.^30^ While PDI is also prone to form large crystalline domains and excimers, which reduces exciton diffusion lengths,^28,31^ recent work shows that PDI derivatives can be tailored to control domain morphology and thereby achieve large increases in OPV efficiency.^28^ Finally, PDI was experimentally demonstrated to have a higher barrier for charge separation in bulk heterojunctions than fullerenes of similar electron affinity.^32^

**Fig. 1:**
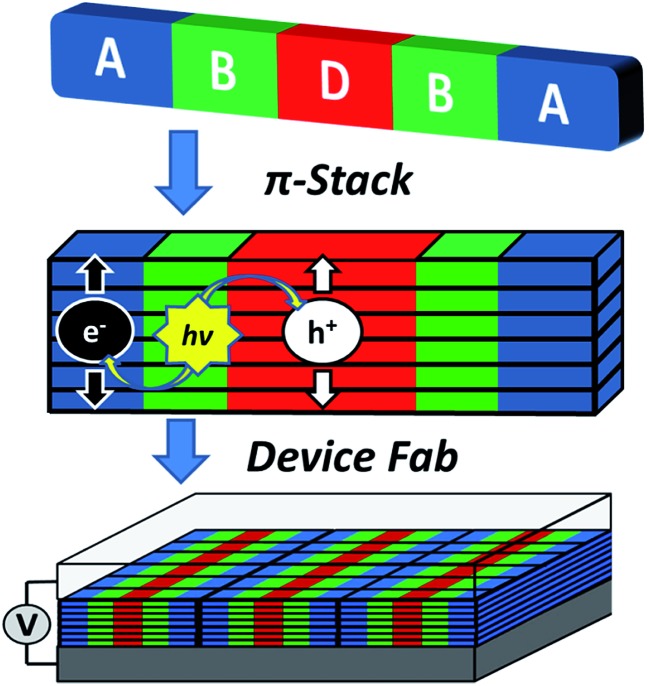
Cartoon depicting the self-assembly of a covalent donor–bridge–acceptor (D–B–A) system to control charge separation and form segregated electron and hole conducting pathways.

**Chart 1:**
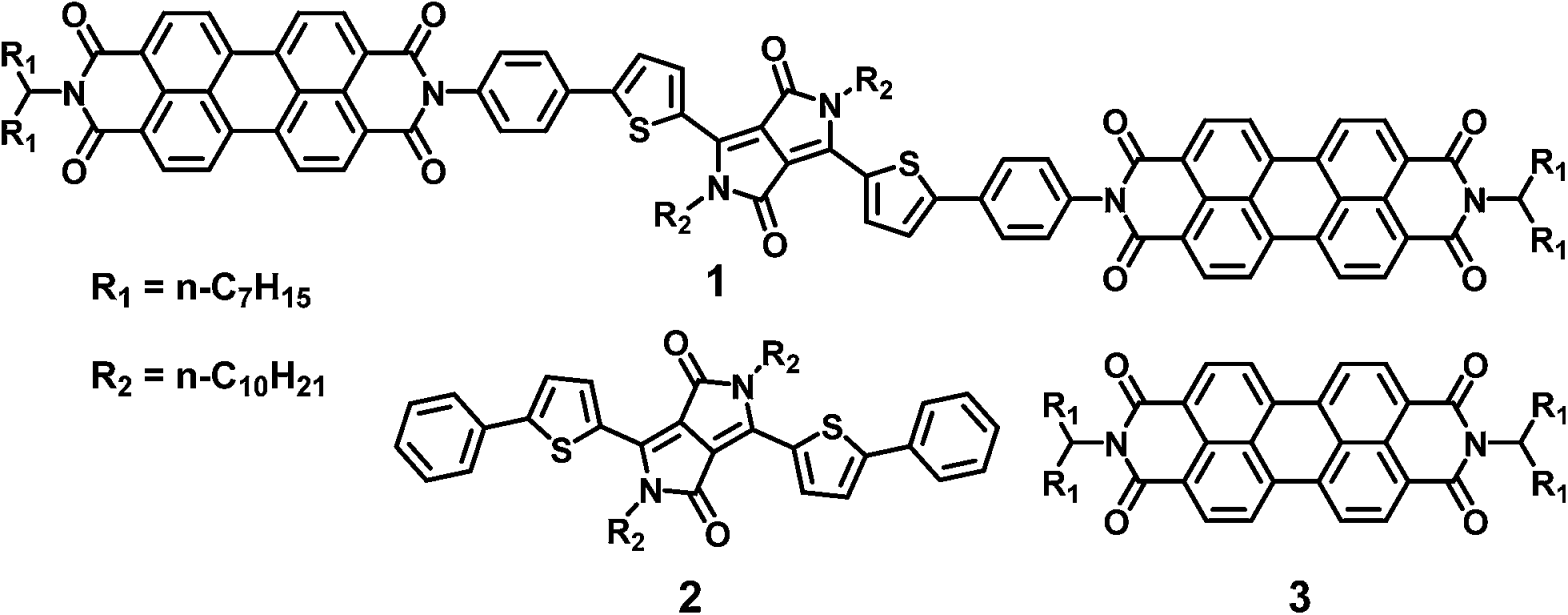
Molecules referenced in this paper.

**Fig. 2:**
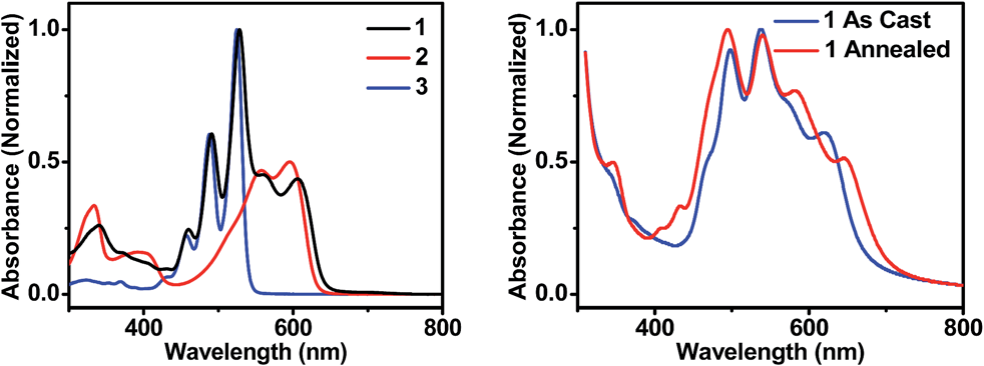
UV-visible absorption spectra of molecules **1**, **2**, and **3** in toluene solution (left), and of thin films of **1** that are unannealed and CH_2_Cl_2_ vapor annealed for 10 minutes (right).

The above constraints are potentially circumvented by using covalent donor–acceptor systems where electron transfer rates can be more easily controlled. In particular, systems can be designed where the molecules self-assemble so that the donor and acceptor domains are segregated into well-defined hole and electron charge conduits, respectively (Fig. 1).^33–35^ Although there are examples of OPVs based on active layers consisting of covalently-connected donor–acceptor systems, some of which have PCEs approaching 2%, there have been few investigations into the photophysics of such systems.^36^ In this contribution we focus on understanding the photophysics of charge separation in thin solid films of donor–acceptor systems by probing the behavior of a diketopyrrolopyrrole (DPP) donor covalently attached to two PDI acceptors, 2,5-bis(1-decyl)-3,6-bis(5-(4-*N*′-1-heptyloctyl-3,4,9,10-perylenediimide-*N*-phenyl)thiophene-2-yl)-2,5-dihydropyrrolo[3,4-*c*]pyrrole-1,4-dione (**1**, PDI–DPP–PDI; Chart 1). Based on the well-established aggregation properties of PDI, the two PDIs within PDI–DPP–PDI are expected to aid the assembly of the monomers into ordered structures similar to that depicted in Fig. 1.^37–39^ Most successful donor materials that have been previously used in OPVs are designed so that their energy levels pair well with fullerene acceptors. Since PDI has a reduction potential similar to that of fullerenes, and this potential is easily tuned by synthetic modification, the energy levels of known successful donor materials also pair well with properly modified PDI acceptors.^40^ In the present case, DPP was chosen as the donor because it has been proven to be an efficient OPV donor, and strongly absorbs visible light at wavelengths complementary to those of PDI (Fig. 2).^41^

The DPP donor in **1** is separated from each PDI acceptor by a single phenyl spacer resulting in a DPP–PDI donor–acceptor dyad in which DPP and PDI are electronically coupled sufficiently well to carry out ultrafast photodriven charge separation.^13^ In fact, such behavior has been observed between PDI and DPP in other systems. In one case, efficient fluorescence quenching, which was attributed to charge transfer, was observed in a DPP polymer donor end-capped by PDI through a covalent attachment similar to that used in **1**.^42^ In a second system, an donor–acceptor–donor structure, formed by hydrogen bonding PDI to two DPP molecules, self-assembles into helical stacks in toluene.^43^ Transient absorption spectroscopy shows that there is rapid charge separation in the assembled system; however, these studies were performed exclusively in solution. If such molecules are to be used in devices, their solid state behavior must be understood. By comparing the photophysics of **1** (Chart 1) in solution to its behavior in thin solid films, it can be determined whether controlling the primary charge separation dynamics using covalent donor–acceptor systems, combined with self-assembly of favorable solid state morphologies, can be used to produce the long-lived free charge carriers necessary for efficient OPV device performance.

## Results and discussion

### Steady-state photophysics and structural studies

In order to maximize sunlight to electricity conversion in an OPV, active layer materials must absorb a large portion of the solar spectrum and the energy levels of the electron donors and acceptors must be situated so that charge transfer is favorable.^44^ The DPP electron donor and the PDI electron acceptor meet these criteria. Unlike fullerenes, PDIs strongly absorb visible light and are easily modified synthetically. PDIs can also have reduction potentials comparable to those of fullerenes and are both inexpensive and air-stable, rendering them ideal candidates for OPV acceptors. DPP has a lower excited singlet state energy than PDI, so it absorbs further to the red, yielding a donor–acceptor system that strongly absorbs over much of the visible spectrum (Fig. 2A). Both PDI and DPP exhibit fluorescence quantum yields near unity in solution (Table 1), which shows that there are no efficient pathways for excited state quenching. The very low (0.1%) fluorescence quantum yield of **1** is attributed to efficient formation of PDI–DPP^+^˙–PDI^–^˙ following photoexcitation (see below).

**Table 1:** Electrochemical and Optical Data

Molecule	*E*_ox_ ^*a*^ (V)	*E*_red_ ^*a*^ (V)	*λ*_abs_ ^*b*^ (nm)	*λ*_em_ ^*b*^ (nm)	*E*_S_ (eV)	*Φ*_F_
1	0.92	–0.58	606	676	1.94	0.001
2	0.87	–1.12	596	674	1.96	0.99
3	1.65	–0.52	525	533	2.35	0.97

^*a*^Oxidation and reduction potentials were measured in 1 mM solutions in CH_2_Cl_2_ with 100 mM TBAPF_6_ as supporting electrolytes for molecules **2** and **3**. Electrochemistry for molecule **1** was measured in CHCl_3_ because of its poor solubility in CH_2_Cl_2_. Potentials were calibrated using a Fc/Fc^+^ internal standard and are reported *vs.* SCE.

^*b*^Absorbance and fluorescence values were measured in toluene solutions and the peak wavelengths are reported.

The sum of the DPP and PDI absorption spectra in model compounds **2** and **3**, respectively, in the ratio of 1 : 2, closely matches the spectrum of **1**, which confirms that PDI and DPP are acting as largely independent chromophores. This is a consequence of the fact that the PDI HOMO and LUMO nodal planes lie along its N–N axis, where the DPP and PDI are linked. The electrochemical properties of molecules **1–3** are summarized in Table 1. That the redox potentials of PDI and DPP within **1** are essentially unchanged from those of the model compounds further supports the assertion that the constituent chromophores are weakly coupled electronically. Since the electronic interaction between PDI and DPP is essentially unchanged by the covalent linkage, a comparison between the photophysics of **1** in solution and in a film allows a direct evaluation of how the photoinitiated charge transfer dynamics differ. Covalent attachment of the chromophores also gives some control over the film morphology because it guarantees that every donor is positioned directly next to an acceptor, minimizing complications arising from exciton diffusion.

The steady-state absorption spectrum of a spin-coated thin film of **1** on glass is shown in Fig. 1B. The spectrum is slightly broader than that in solution, and the vibronic bands are broadened due to aggregation in the solid state. Of particular interest is the increased oscillator strength of the second PDI vibronic band relative to the first vibronic band. This difference has been attributed to H-aggregation in PDI and suggests that in the film the PDI molecules assemble cofacially, which is required to form conductive pathways.^45–47^ The enhancement of the second vibronic band is likely greater than it appears since the entire PDI absorption spectrum overlaps with the blue edge of the DPP absorption (Fig. 1A). Upon solvent vapor annealing (SVA) the film of **1** in a saturated CH_2_Cl_2_ atmosphere, the spectrum is further broadened and the second PDI vibronic absorbance is further enhanced, suggesting that there is a higher degree of intermolecular coupling between PDI chromophores in the annealed film.

**Fig. 3:**
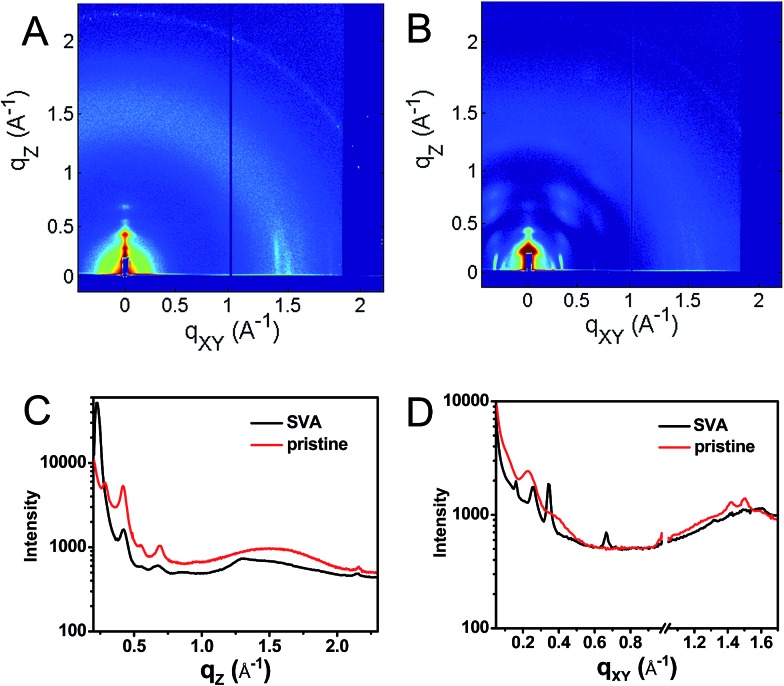
GIWAXS of a pristine (A) film of **1** and a film which has been solvent vapor annealed in CH_2_Cl_2_ for 10 min (B) as well as vertical (C, *q*_*xy*_ = 0) and horizontal (D, *q*_*z*_ = 0) line cuts of the annealed (black) and unannealed (red) films.

This hypothesis was verified by grazing incidence X-ray scattering experiments performed at the Advanced Photon Source at Argonne National Laboratory. Line cuts of the scattering images (Fig. 3) show a redistribution of scattering intensity from the broad, radial bands (0.39 and 1.5 Å^–1^) indicative of amorphous character, to narrower, intense peaks (0.22, 0.26, and 0.34 Å^–1^) characteristic of highly crystalline films. The molecular orientation is also impacted by solvent vapor annealing. The emergence of the peak at 0.22 Å^–1^ corresponds to parallel alignment of the long molecular axes with the substrate normal. This assignment is made based on the correspondence between one-half the molecular length (56 Å/2 = 28 Å) of the geometry-optimized molecular structure (B3LYP/6-31G*) and the strongest *d*-spacing (2π/0.22 Å^–1^ = 28.6 Å). The GIWAXS results evidence greater order in the annealed film and are also consistent with the changes observed in its UV-Vis absorption spectrum *versus* the unannealed film. The orientation of **1** in the SVA film is perpendicular to the most desired orientation for OPVs shown in Fig. 1, likely a consequence of the hydrophilic nature of the glass surface. Nevertheless, the formation of free charge carriers can be readily assessed in these ordered layers. Reorienting the layers of **1** so that they π-stack perpendicular to the surface would require, at a minimum, modifying the glass substrate to make it hydrophobic.

The free energy for electron transfer in bulk heterojunctions is commonly approximated by the LUMO–LUMO offset, or difference in reduction potentials, of the donor and acceptor. The offset in **1** is 0.6 eV and should be sufficient since charge separation is commonly quite rapid in systems with a LUMO–LUMO offset greater than 0.3 eV.^48,49^ The ion pair free energy for electron transfer in solution can be approximated using electrochemical redox potentials and the Weller equation which is based on the Born dielectric continuum model, and takes into account coulombic interactions with the two ions and their solvation energies:1
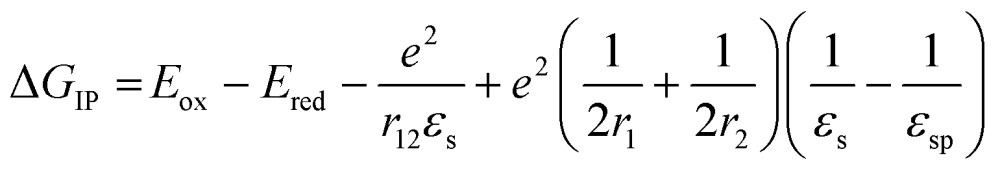
where Δ*G*_IP_ is the ion pair energy; *E*_ox_ and *E*_red_ are the one-electron oxidation and reduction potentials of the donor and acceptor, respectively; *r*_12_ is the distance between the separated charges; *r*_1_ and *r*_2_ are the ionic radii; *ε*_s_ is the dielectric constant of the solvent of interest; and *ε*_sp_ is the dielectric constant of the solvent in which the redox potentials are measured.^12,50^ The oxidation potential of **2** in CH_2_Cl_2_ is 0.87 V *vs.* SCE and the reduction potential of **3** in CH_2_Cl_2_ is –0.52 V *vs.* SCE. Using these values, and interatomic distances based on an optimized structure of **1** (B3LYP/6-31G*), the ion pair energy for this system in toluene (*ε*_s_ = 2.38) is calculated to be 1.70 eV. Since this is lower than the singlet excited state energies of both **2** (2.04 eV) and **3** (2.34 eV), charge separation is expected to be favorable with Δ*G*_IP_ = –0.34 eV at a minimum.

### Spectroelectrochemistry

**Fig. 4:**
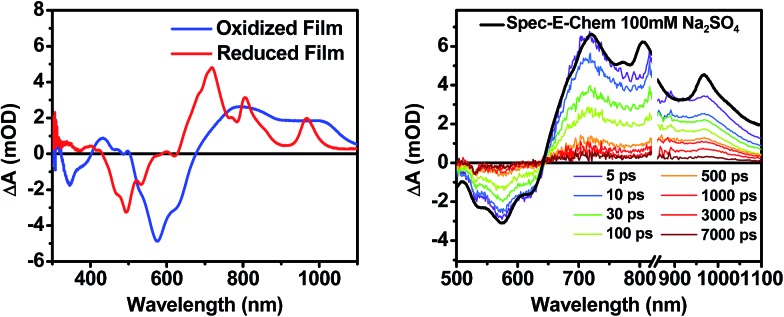
(Left) A comparison of spectroelectrochemical difference spectra of **1** electrochemically oxidized and reduced in a film on ITO. (Right) A comparison of the sum of the reduced and oxidized species spectra to the fsTA spectrum of an unannealed film of **1**.

The DPP^+^˙ and PDI^–^˙ UV-vis spectra of **1** in annealed thin films as well as the DPP^+^˙ spectrum of **2** were investigated by spectroelectrochemistry, to provide radical ion spectra for interpreting the transient absorption data (Fig. 4A and S3†). The PDI^–^˙ spectrum is well established in the literature.^51^ The thin film PDI^–^˙–PDI difference spectrum has absorption maxima at 700, 800, and 950 nm, which are consistent with what is reported for PDI^–^˙ in solution.^51^ There is also ground state bleaching that would be indicative of H-aggregated PDI. The bleach of the PDI 0–1 vibronic band in the PDI^–^˙–PDI difference spectrum is even more pronounced than the 0–1 absorption in the ground state spectrum because it is not influenced by the DPP spectrum, which is subtracted in the difference spectrum. In the DPP^+^˙–DPP difference spectrum, the DPP ground state bleach is clearly visible at 600 nm and is accompanied by broad DPP^+^˙ absorptions at 420, 800, and 1000 nm. These features are consistent with the DPP^+^˙ features observed by spectroelectrochemistry of **2** in solution (Fig. S3†).

### Transient absorption spectroscopy in solution

**Fig. 5:**
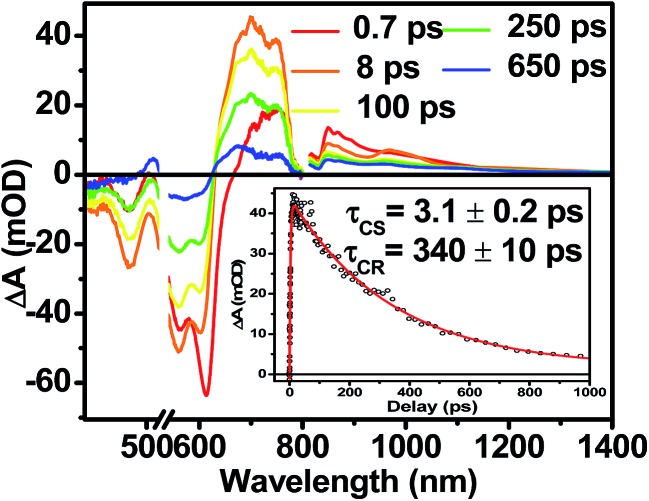
fsTA spectra of **1** in toluene. Inset shows the kinetics and fit of the data at 700 nm.

The photophysics of **1–3** in toluene solution were studied using fsTA spectroscopy. Toluene was chosen as the solvent because of its low dielectric constant (*ε*_s_ = 2.38), which allows more direct comparison to the film data because the dielectric constant in similar organic thin films is typically ∼3.^17,52^ Molecules **2** and **3** display transient spectra consisting of ground state bleaching, stimulated emission, and a photoinduced absorbance, which is assigned to singlet excited state absorption at 800 nm in **2** and 700 nm in **3** (Fig. S4 and S6†). Global fitting of the transient data show that the singlet excited states of **2** and **3** decay monoexponentially in *τ* = 3.0 ns and 3.8 ns, respectively. These long monoexponential decays combined with the high fluorescence quantum yields of both **2** and **3** simplify the interpretation of the spectra of **1** because the data show that there are no intrinsic fast pathways for excited state quenching of ^1*^DPP and ^1*^PDI. This means that any fast kinetics observed in the fsTA spectra of **1** are due to interactions between the donor and acceptor chromophores. At short times, the fsTA spectra of **1** following photoexcitation at 532 nm (Fig. 5) are consistent with instrumentation-limited formation of ^1*^DPP as indicated by ground state bleaching, stimulated emission at 610 nm, and broad excited state absorption at longer wavelengths. The stimulated emission decay is accompanied by the simultaneous appearance of 700 nm PDI^–^˙ and 750 nm DPP^+^˙ absorptions in *τ* = 3.1 ± 0.2 ps. These spectral changes and the fact that the appearance of the DPP^+^˙–PDI^–^˙ radical ion pair (RP) is nearly 100 times faster than ^1*^DPP decay indicate that charge separation is quantitative. At later times, DPP^+^˙–PDI^–^˙ recombines in *τ* = 340 ± 10 ps. The charge recombination kinetics in solution are monoexponential, suggesting that the molecule is disaggregated in solution and that recombination is dominated by a single process. The observed rapid charge separation and recombination for **1** in solution is consistent with the degree of donor–acceptor electronic coupling resulting from having only a single phenyl spacer between DPP and PDI.^13^

### Transient absorption spectroscopy in thin solid films

**Fig. 6:**
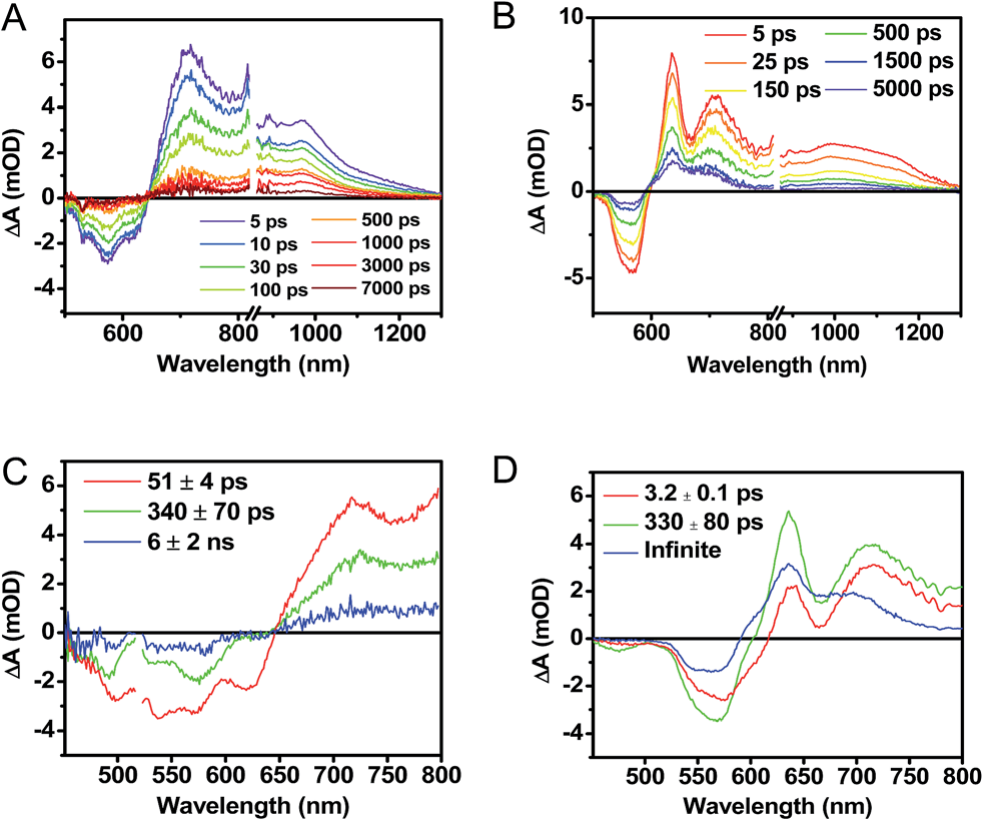
fsTA spectra of an as cast, unannealed thin film of **1** (A), and a film that was solvent vapor annealed for ten minutes with CH_2_Cl_2_ (B). Singular value decomposition (SVD) and global fitting gave decay associated spectra for the unannealed (C) and annealed (D) films.

Following photoexcitation, the transient absorption spectra of the unannealed film of **1** (Fig. 6A) exhibit spectral features indicative of DPP^+^˙–PDI^–^˙ formation that appear in *τ* < 250 fs. This fast charge separation is followed by multiexponential charge recombination (Fig. 6C), which is attributed to a distribution of molecular environments. Singular value decomposition (SVD) and global fitting of the data show that the majority of the DPP^+^˙–PDI^–^˙ population decays in *τ* = 51 ps (55%). This rapid charge separation and recombination process most likely involves direct intermolecular interactions between π-stacked DPP/PDI pairs in the disordered film because it is much faster than intramolecular DPP^+^˙–PDI^–^˙ charge recombination for **1** in solution. The next largest DPP^+^˙–PDI^–^˙ population recombines with a time constant (*τ* = 340 ps, 35%) similar to that obtained in solution, which suggests that this population undergoes intramolecular charge recombination. Finally, a small population of DPP^+^˙–PDI^–^˙ decays with a longer time constant (*τ* ∼ 6 ns, 10%), which is much faster than the free charge carrier recombination lifetimes typically observed in BHJ films.^53^ These results indicate that there is very little dissociation of the initially-formed DPP^+^˙–PDI^–^˙ RP to produce free charge carriers in the disordered film.

**Fig. 7:**
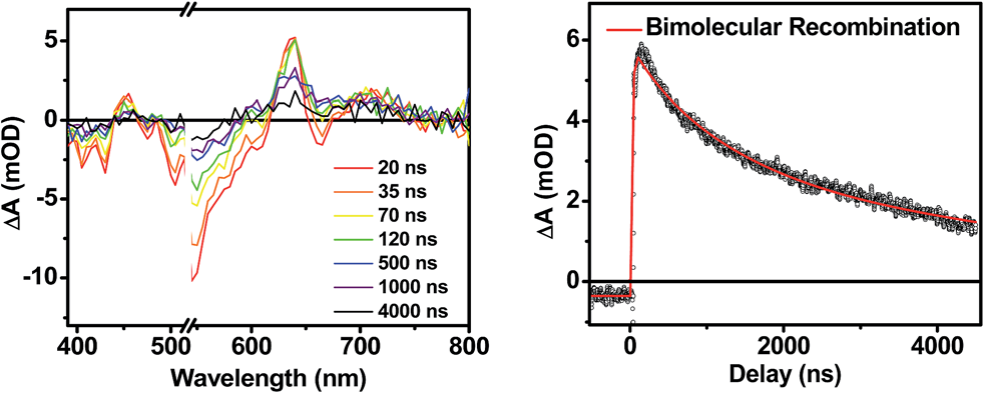
Nanosecond transient spectra of an annealed film of **1** (left) and its transient kinetic trace at 645 nm (right). The nsTA data (black points) is fit using a bimolecular decay (red line).

The transient absorption spectra of the solvent vapor annealed (SVA) films of **1** (Fig. 6B) differ somewhat from those observed for the unannealed films as a result of the preferential orientation of the molecular long axes along the substrate normal as discussed above (Fig. 3). The most obvious difference is the growth of a sharp positive feature at 635 nm, accompanied by broadening of the near-infrared absorbance. Singular value decomposition (SVD) and global fitting give decay-associated spectra (Fig. 6D) which reveal that the 635 and 715 nm bands appear in *τ* = 3.2 ps. This time constant is identical to that observed for charge separation in solution and is thus assigned to intramolecular charge separation. The 715 nm band blue shifts to 695 nm in *τ* = 330 ps (50%), which matches the intramolecular DPP^+^˙–PDI^–^˙ charge recombination time constant observed in the unannealed film and in solution. This component is assigned to charge recombination of DPP^+^˙–PDI^–^˙ pairs that are trapped on the individual molecules of **1** by coulombic attraction or because local disorder prevents further charge separation to free ions. However, unlike the unannealed film, the SVA film of **1** has a substantial (30%) kinetic component having *τ* ≫ 6 ns with broadened absorption bands, which are assigned to free charge carriers in the film. This long decay time of the charge carriers exceeds the maximum pump/probe delay time of the fsTA apparatus, so that nsTA was used to obtain the decay kinetics. The decay kinetics of the 635 nm peak can be fit to a single bimolecular recombination rate constant of *k*_CR_ = (8.5 ± 0.1) × 10^7^ M^–1^ s^–1^ (Fig. 7), which is consistent with long-lived free charge carriers that diffuse within the film and then recombine. The higher degree of molecular order in the annealed film greatly increases the number of segregated, π-stacked PDI–DPP–PDI molecules (Fig. 1) at the expense of directly π-stacked DPP/PDI pairs. At this point we cannot determine whether the electrons and hole are confined within single segregated π-stacks, but we expect that inter-stack PDI–PDI and or DPP–DPP hopping will be considerably slower than intra-stack hopping.

It is useful to note here that these transient spectral changes may also have contributions from excimer state formation and/or triplet states formed by charge recombination or singlet exciton fission. Any of these processes could result from the increased order in the solvent annealed film observed by GIWAXS (Fig. 3). Considering the first case, it is unlikely that the observed species is an excimer state because it is not observed in films of either **2** or **3**. Also, the microsecond lifetime of the long-lived species is inconsistent with what has been observed for excimers, for example, PDI excimer states live no longer than about 30 ns.^54–56^ In contrast, free charge carrier lifetimes have been observed in the microsecond and even millisecond regime, which is consistent with the observed lifetime in the SVA film of **1**.^57,58^

**Fig. 8:**
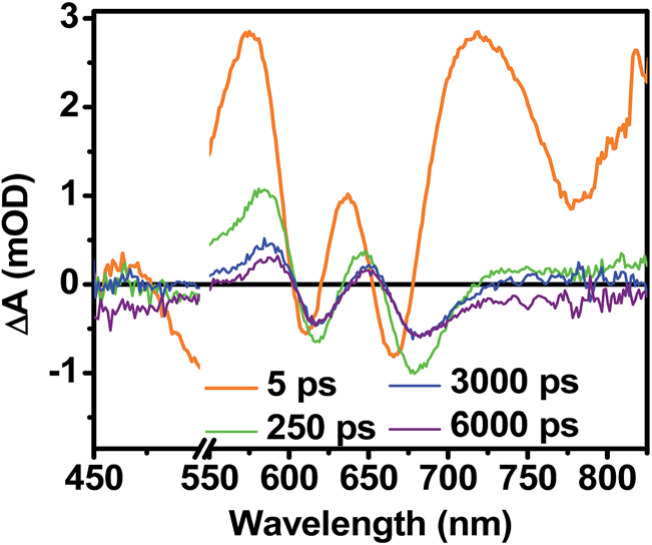
FsTA spectra of a film of **2** that has been solvent vapor annealed for 10 minutes in CH_2_Cl_2_ showing positive absorptions at 580 and 650 nm resulting from ^3*^DPP formation.

While triplet formation for both DPP and PDI is very slow in solution, fast triplet formation in the solid film could result from singlet exciton fission, which has recently been shown to occur with *τ* = 180 ps in a PDI derivative having a slip-stacked geometry in the solid state.^59^ In order to further examine the possibility of triplet formation, the lowest energy triplet state of **1** was computed and found to reside on DPP rather than PDI (Fig. S8†). The calculated triplet energy of ^3*^DPP is 1.01 eV, while the experimentally measured value for ^3*^PDI is 1.28 eV.^59^ Moreover, the calculated triplet energy of ^3*^DPP in **2** closely matches that in **1**. Since the lowest excited triplet state of both **1** and **2** is ^3*^DPP, if photoexcitation of **1** produces a triplet state, its transient spectrum should resemble the triplet–triplet absorption spectrum of **2**. FsTA spectroscopy was performed on a thin film of **2** annealed under the same conditions as the films of **1** (Fig. 8). Global fitting of the data gives an initial spectrum that appears within the instrument response time, which is attributed to ^1*^DPP and decays in 30 ps to give a long-lived spectrum (*τ* ≫ 6 ns) that is assigned to ^3*^DPP in **2**. This ultrafast triplet formation is most likely a result of singlet exciton fission in films of **2**, which will be discussed in a future publication. However, charge separation in the SVA film of **1** (*τ* = 3.2 ps) outcompetes DPP singlet exciton fission (*τ* = 30 ps), and the observed ^3*^DPP transient spectrum observed in **2** is dissimilar to that of the SVA film of **1**, showing that the long-lived species observed following photoexcitation of **1** is not ^3*^DPP. Note also that the long-lived species observed in the SVA films of **1** is not a linear combination of the ^3*^DPP and DPP^+^˙–PDI^–^˙ spectra. Moreover, since all of the features in the long-lived spectrum of **1** decay with the same time constants, in all likelihood they correspond to a single species.

### Magnetic field effects on charge recombination

**Fig. 9:**
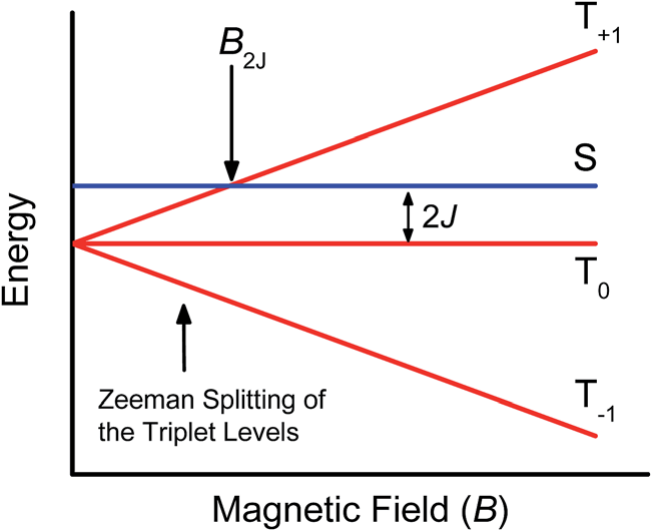
Schematic of radical ion pair energy levels as a function of magnetic field for *J* > 0.

Further confirmation that the long-lived transient species observed in the SVA film of **1** results from a charge separated state comes from magnetic field effect (MFE) measurements. Following charge separation, the initially-formed spin-correlated singlet RP, ^1^(DPP^+^˙–PDI^–^˙) undergoes electron-nuclear hyperfine coupling-induced radical-pair intersystem crossing (RP-ISC)^60,61^ to produce the triplet RP, ^3^(DPP^+^˙–PDI^–^˙). Application of a magnetic field results in Zeeman splitting of the ^3^(DPP^+^˙–PDI^–^˙) triplet sublevels, which are best described by the |*T*_+1_, |*T*_0_, and |*T*_–1_ eigenstates that are quantized along the applied magnetic field direction, while ^1^(DPP^+^˙–PDI^–^˙) is described by the |*S* eigenstate (Fig. 9).^60,62^ The energy difference between |*S* and |*T*_0_ is the spin–spin exchange interaction 2*J*, which is assumed to be isotropic. In general, the subsequent formation of products from the singlet and triplet RPs is spin selective; *i.e.*, ^1^(DPP^+^˙–PDI^–^˙) will recombine to the singlet ground state or charge separate further to give free charge carriers, while ^3^(DPP^+^˙–PDI^–^˙) will recombine to the neutral triplet state ^3^(DPP–PDI), provided that the energy of either ^3*^DPP or ^3*^PDI is below that of ^3^(DPP^+^˙–PDI^–^˙), or once again will yield free charge carriers.^13^ Since the energies of ^3*^DPP and ^3*^PDI are both below the energy of ^3^(DPP^+^˙–PDI^–^˙), the triplet charge recombination channel is potentially accessible in **1**.

**Fig. 10:**
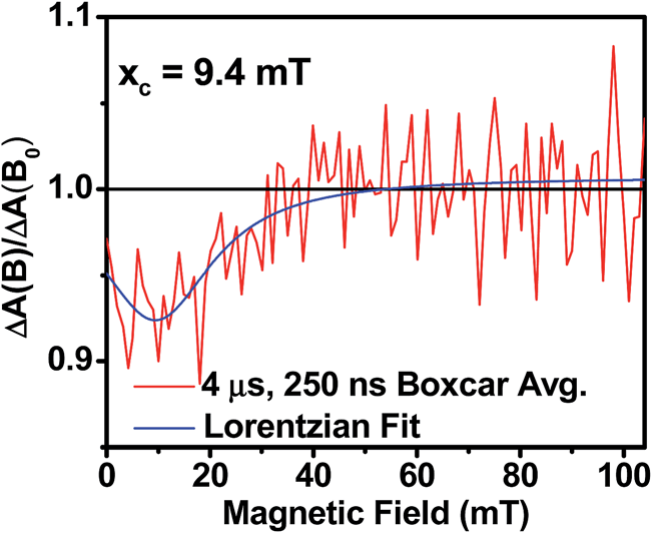
Relative transient absorption intensity of **1** films *vs.* magnetic field measured at 635 nm and 4.0 μs after a 7 ns, laser pulse.

As the magnetic field strength, *B*, is increased, level crossing of the *S* and *T*_+1_ (*J* > 0) or *T*_–1_ (*J* < 0) energy levels enhances singlet–triplet mixing when *B* = 2*J* (Fig. 9). If product formation is more facile *via* the triplet channel than the singlet channel, the increase in triplet-derived product yield when *B* = 2*J* is usually manifested as a resonance in a plot of triplet-derived product yield *vs. B* or as an anti-resonance in the corresponding plot of the RP precursor yield. For the SVA film of **1**, the magnetic field dependence of the transient absorption at 635 nm resulting from DPP^+^˙ and PDI^–^˙ obtained 4 μs after the laser pulse exhibits an *anti-resonance* at 2*J* = 9.4 mT (Fig. 10). Note that RP-ISC does not result in changes in the RP optical absorption spectrum, *i.e.*, ^1^(DPP^+^˙–PDI^–^˙) and ^3^(DPP^+^˙–PDI^–^˙) have the same transient absorption spectra. The observed ∼8% loss of free charge carrier population when *B* = 2*J* is attributed to formation of small amounts of ^3*^DPP–PDI by charge recombination of ^3^(DPP^+^˙–PDI^–^˙). Once the triplet channel is accessible when *B* = 2*J*, the reaction ^3^(DPP^+^˙–PDI^–^˙) → ^3*^DPP–PDI becomes more kinetically competitive with charge recombination of the free carriers to ground state *via* the singlet channel. This is largely a consequence of the fact that the free energy for the triplet charge recombination reaction ^3^(DPP^+^˙–PDI^–^˙) → ^3*^DPP–PDI is –0.5 eV, placing it in the Marcus normal region (fast reaction), while that of the singlet charge recombination ^1^(DPP^+^˙–PDI^–^˙) → DPP–PDI is –1.5 eV, placing it in the Marcus inverted region (slow reaction).

The resonance at 2*J* is also much broader than what is commonly observed in solution, which most likely results from a distribution of distance and orientation dependent RP magnetic interactions within the film. For example, the distance dependence of *J* = *J*_0_e^–*β*(*r*–*r*_0_)^, where *J*_0_ is defined at the van der Waals contact distance *r*_0_ (3.5 Å) and *r* is the RP distance. Since *J*_0_ = 5.0 × 10^5^ mT and *β* = 0.6 for π-stacked PDI-based donor–acceptor systems,^35^ and the experimental *J* = 4.7 mT, the average electron–hole pair distance is 23 Å. It is well known that 2*J* ∝ *V*_CR_^2^,^13^ where *V*_CR_^2^ is the electronic coupling matrix element for charge recombination. Moreover, electron transfer theory shows that the charge recombination rate constant *k*_CR_ ∝ *V*_CR_^2^,^13^ so that the small 2*J* = 9.4 mT ≅ 0.01 cm^–1^ value and the long RP lifetime are both consistent with weak electronic coupling between DPP^+^˙ and PDI^–^˙ in the solid. The microsecond lifetime observed in annealed films of **1** is significantly longer than carrier charge collection time constants measured by transient photocurrent experiments.^63^ This suggests that the charge carriers in an annealed film of **1** live long enough to be extracted from an OPV incorporating **1** as the active layer.

## Conclusions

Long-lived DPP^+^˙ and PDI^–^˙ free charge carriers form *via* a spin-correlated radical ion pairs in annealed films of PDI–DPP–PDI, **1**, as detected by transient absorption spectroscopy and magnetic field effect experiments. In marked contrast, while charge separation in the same molecule is also observed in unannealed films, it is inefficient and most of the charge-separated state population recombines in less than a nanosecond, arguing that geminate recombination dominates in unannealed films. The longer RP lifetime in the annealed films suggests that the charge carriers are stabilized by dissociation into free charge carriers. The charge recombination kinetics are fit well by a bimolecular rate equation, which suggests that the charge carriers are in fact separating and diffusing through the film.

The long-lived charge-separated state observed in annealed films of molecule **1** suggests that charge carrier generation in the solid state is possible even in donor–acceptor systems having relatively fast solution phase charge recombination time constants. This means that covalent donor–acceptor systems with non-fullerene acceptors can be designed with high charge separation quantum yields. These materials are potentially useful as active layer materials in OPVs. Devices using **1** as the active layer are currently under investigation.

## Experimental methods

### Synthesis

**Scheme 1:**
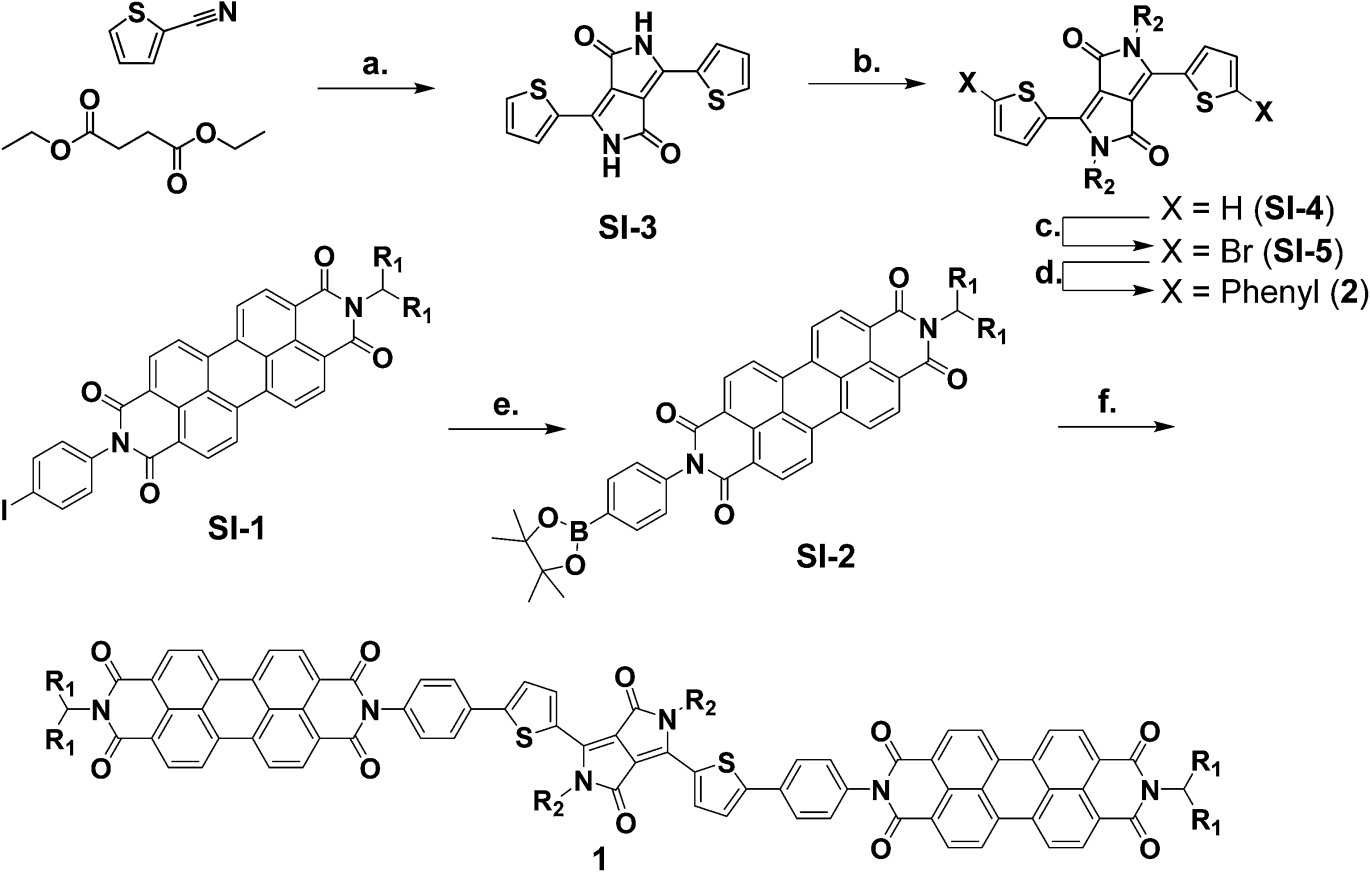
Synthesis of molecules **1–2**.^*a a*^(a) *t*-Amyl alcohol, KOtBu, 120 °C, 34% (b) DMF, K_2_CO_3_, 1-bromodecane, 120 °C, 85% (c) DCM, NBS, RT, 59%, (d) toluene, EtOH, H_2_O, K_2_CO_3_, phenylboronic acid, Pd(PPh_3_)_4_, 60 °C, 95%, (e) DMF, bis(pinacolato)diboron, KOAc, Pd(dppf)Cl_2_, 100 °C, 98%, (f) toluene, EtOH, H_2_O, K_2_CO_3_, **SI-5**, Pd(PPh_3_)_4_, 65 °C, 64%.

The syntheses of molecules **1** and **2** are outlined in Scheme 1. The synthetic details and characterization data are presented in the ESI. Compounds **3** and **SI-1** were prepared according to known literature procedures.^64,65^

### Sample preparation

Solution samples were prepared in toluene and passed through a 300 μm syringe filter prior to measurement. Due to the poor solubility of **1** in toluene, the solution was sonicated for 30 min at 50 °C prior to filtration. Film samples of **1** were spin-coated from a 20 mg mL^–1^ solution in CHCl_3_ at 1000 rpm on glass cover slips. Annealed samples were placed in a glass Petri dish above a reservoir of CH_2_Cl_2_ that had been allowed to equilibrate for 30 min prior to annealing. The samples were removed after 10 min and dried under reduced pressure. The films used for transient absorption spectroscopy were brought into a glovebox and placed face-down on a glass slide. The edges of the cover slip were sealed to the glass slide using UV-curing epoxy.

### Electrochemistry

Cyclic voltammetry and differential pulse voltammetry were performed on 1 mM solutions of **1** in dry CHCl_3_ and **2–3** in dry CH_2_Cl_2_ with 100 mM tetrabutylammonium hexafluorophosphate as the supporting electrolyte. All measurements were done using a platinum disc working electrode, a platinum wire counter electrode, and a silver wire quasi-reference electrode (AgQRE). All redox potentials were referenced to ferrocene as an internal standard and are reported *vs.* SCE.

Spectroelectrochemistry was performed on molecule **1** to acquire its radical ion pair spectrum. Molecule **1** was spin coated onto an ITO working electrode which was submerged in a 100 mM aqueous solution of sodium sulfate supporting electrolyte with a platinum mesh counter electrode and a AgQRE. The spectrum of the oxidized species was obtained at a potential of 1.0 V *vs.* AgQRE and the spectrum of the reduced species was obtained at –0.8 V *vs.* AgQRE. Both the oxidation and reduction processes in the film were confirmed to be reversible by poising the potential at 0 V *vs.* AgQRE until the original spectrum was recovered.

### Steady-state spectroscopy

Steady-state absorbance spectra were measured using a Shimadzu UV-1800 spectrometer. Solution spectra were measured in toluene in a 2 mm quartz cuvette. Film samples were measured directly on their glass substrates. Fluorescence spectra were measured using a Photon Technology International photon-counting spectrofluorimeter. Solution phase measurements were carried out in a 1 cm glass cuvette in a dilute CH_2_Cl_2_ solutions. Film measurements were carried out directly on the films, which were placed at a 20° angle to the excitation beam.

### Femtosecond transient absorption spectroscopy (fsTA)

The details of the transient absorption experiments have been reported previously.^59^ Briefly, for visible and near-infrared fsTA, samples were excited at 532 nm with 0.5 μJ per pulse at 1 kHz. Solution samples were dissolved in toluene to an optical density of between 0.5 and 0.7 at the excitation wavelength in a 2 mm pathlength quartz cuvette and the pump and white-light continuum probe pulses were focused to a 200 μm diameter spot size at the sample. The excitation and probe pulses for film samples were focused to 1 mm diameter spot sizes in order to reduce the excitation density.

### Nanosecond transient absorption spectroscopy (nsTA)

The film samples were evacuated to 10^–3^ Torr in a Janis VPF-100 cryostat. Nanosecond transient absorption experiments were performed by exciting the sample with 7 ns, 1.8 mJ, 532 nm pulses using the frequency-tripled output of a Continuum Precision II 8000 Nd-YAG laser pumping a Continuum Panther OPO. The probe pulse, generated using a xenon flashlamp (EG&G Electro-Optics FX-200), and pump pulse were overlapped on the sample with the pump beam focused to a spot size slightly larger than that of the probe beam. Kinetic traces were observed from 390–800 nm every 5 nm using a 416 nm long-pass filter (above 420 nm), a monochromator, and photomultiplier tube (Hamamatsu R928) with high voltage applied to only 4 dynodes. Kinetic traces were recorded with a LeCroy Wavesurfer 42Xs oscilloscope interfaced to a data system using a custom Labview program (Labview v. 8.6.1). Spectra were constructed from the single wavelength kinetic traces taken every 5 nm. Each kinetic trace is representative of an average of 150 shots. To increase the signal-to-noise ratio of the spectral profiles, 5–10 ns segments of data are averaged, and the median time is reported as the time of the spectral slice.

### Transient absorption spectroscopy data analysis

Single wavelength (or frequency) kinetic analyses were performed using a Levenberg–Marquardt nonlinear least-squares fit to a sum of exponentials convoluted with a Gaussian instrument response function. The ns data were also fit using a bimolecular rate function convoluted with the instrument response as described previously.^66^ The three-dimensional data sets of Δ*A vs.* time and wavelength were subjected to singular value decomposition (SVD), and global fitting to obtain the kinetic time constants and their decay-associated spectra using Surface Xplorer software (Ultrafast Systems LLC, Sarasota, FL).

### Magnetic field effect experiments

Magnetic field effect (MFE) experiments were performed using the same pump–probe setup as was used for nsTA. For the MFE experiments, the cryostat was placed between the poles of a Walker Scientific HV-4W electromagnet powered by a Walker Magnion HS-735 power supply. The field strength was measured by a Lakeshore 450 gaussmeter with a Hall effect probe. Both the electromagnet and the gaussmeter were interfaced to the data acquisition computer, allowing measurement and control of the magnetic field to ±1 × 10^–5^ T during data acquisition. To account for any sample degradation that might occur over the course of the experiment (>3 h), Δ*A*(*B* = 0) was collected every three kinetic traces and fit with a linear polynomial. These functions were used to calculate Δ*A*(*B*)/Δ*A*(*B* = 0). The MFE plots shown in Fig. 10 are representative of a 50 ns adjacent-average of Δ*A* at *t* = 4 μs (baselined to –250 ns), taken from 500-shot average kinetic traces at 635 nm and 450 nm. The kinetic traces were measured at magnetic fields from 0–100 mT, in 1 mT steps with a 0.2 mT tolerance.

### GIWAXS measurements

Grazing incidence X-ray scattering (GIWAXS) measurements were obtained at Beamline 8-ID-E of the Advanced Photon Source at Argonne National Laboratory. The X-ray beam (7.35 keV, 1.6868 Å) penetrated the sample at an incident angle of 0.2° in order to maximize film scatter and minimize background scatter from the glass substrate. A Pilatus 1 M detector positioned 204 mm from the sample was used to collect the scattered light. Scattering coordinates are expressed in terms of *q* = 4π sin(*θ*)/*λ*, and can be related to the *d*-spacing by *q* = 2π/*d*. GIXSGUI, a MATLAB-based program, was used to apply pixel efficiency, polarization, flat field, and solid angle corrections to the detector images, as well as to display images and take line cuts.^67^
