# Supplementary material for: Long-lived charge carrier generation in ordered films of a covalent perylenediimide–diketopyrrolopyrrole–perylenediimide molecule
Source: Chem Sci. 2014 Sep 16;6(1):402–11. doi: 10.1039/c4sc02551b (PMC5587985; doi:10.1039/c4sc02551b)
Supplement: Supplementary file 1 [file SC-006-C4SC02551B-s001.pdf]

Electronic Supplementary Material (ESI) for Chemical Science.
This journal is © The Royal Society of Chemistry 2014

Electronic Supplementary Information for

Long-lived Charge Carrier Generation in Ordered Films of a Covalent Perylenediimide-Diketopyrrolopyrrole-Perylenediimide Molecule

Patrick E. Hartnett, Scott M. Dyar, Eric A. Margulies, Leah E. Shoer, Andrew W. Cook, Samuel W. Eaton, Tobin J. Marks*, and Michael R. Wasielewski*

Department of Chemistry and Argonne-Northwestern Solar Energy Research (ANSER) Center, Northwestern University, Evanston, Illinois 60208-3113.

E-mail: m-wasielewski@northwestern.edu; t-marks@northwestern.edu; Fax: +1-847-467-1425; Tel: +1-847-467-1423

Synthesis

N-4-iodophenyl-N'-1-heptyloctyl-3,4,9,10-perylenediimide was synthesized according to reported procedures.[1] Potassium acetate, sodium tert-butoxide, 1-bromodecane, phenylboronic acid, and diethylsuccinate were purchased from Sigma-Aldrich (St. Louis, MO). Thiophene carbonitrile was purchased from Oakwood Chemicals (West Columbia, SC). Pd(dppf)Cl_2 and bis(pinacolato)diboron were purchased from Frontier Scientific (Logan, UT). $Pd(PPh_3)_4$ was purchased from Strem Chemical (Newburyport, MA). Flash chromatography was performed using Sorbent Technologies (Atlanta, GA) silica gel. All solvents were spectrophotometric grade unless otherwise noted. ^{1}H nuclear magnetic resonance spectra were obtained on a Bruker Avance III 500MHz spectrometer. Matrix-assisted laser-desorption-ionization time-of-flight (MALDI-TOF) mass spectra were obtained using a Bruker Daltonics Autoflex III Smartbeam MALDI mass spectrometer in negative ionization mode without matrix for PDI samples and positive ionization mode for DPP samples.

Synthesis of N-(4-(4,4,5,5-tetramethyl-1,3,2- dioxoborolan-2-yl)phenyl)-N'-1-heptyloctyl-3,4,9,10-perylenediimide, PDI-Phenyl Boronic Ester (SI-2). 500mg(0.6mmol) of N-4-iodopheny)-N'-1-heptyloctyl-3,4,9,10-perylenediimide was combined with 315mg(1.2mmol) bis(pinacolato)diboron, 180mg potassium acetate, and 45mg(0.06mmol) Pd(dppf)Cl_2 in a 100mL two necked round bottom flask equipped with a reflux condenser. The flask was evacuated and purged with nitrogen three times and 50mL of dry degassed DMF was injected. The resulting solution was stirred at 100°C for one hour. The reaction mixture was cooled to room temperature, diluted with DCM (50mL) and washed with water (3x100mL). The organic layer was collected and the solvent was removed under reduced pressure. The resulting solid was dissolved in a minimal amount of DCM (3mL) in a 50mL centrifuge tube and the tube was filled with methanol resulting in a bright red precipitate. The precipitate was collected by centrifugation and washed with methanol to give the product as a bright red solid (490mg, 98%). ^{1}H NMR (500 MHz, $CDCl_3$) δ 8.82 – 8.60 (m, 8H), 8.09 – 7.95 (d, J = 7.6 Hz, 2H), 7.42 – 7.31 (d, J = 7.6 Hz, 2H), 5.26 – 5.11 (m, 1H), 2.32 – 2.17 (m, 2H), 1.92 – 1.79 (m, 2H), 1.41 – 1.34 (s, 12H), 1.37 – 1.14 (m, 20H), 0.85 – 0.76 (t, J = 6.2 Hz, 6H). MALDI-TOF (*m/z*) $[M]^-$ = 802.554 (Calcd for $C_{51}H_{55}BN_2O_6$: 802.415)

Synthesis of 2,5-Bis(1-decyl)-3,6-bis(5-(4-PDI-phenyl)thiophene-2-yl)-2,5-dihydropyrrolo[3,4-c]pyrrole-1,4-dione, PDI-DPP-PDI (1). 110mg(0.137 mmol) of **SI-2**, 50mg(0.068 mmol) of DPP-Br_2, and 80mg(0.58 mmol) of potassium carbonate were mixed with 10mL toluene, 1mL absolute ethanol, and 1mL deionized water. The resulting mixture was bubbled with nitrogen for 30 minutes and 10mg(0.008 mmol) of Pd(PPh_3) was added. The reaction mixture was bubbled with nitrogen for an additional 15 minutes and heated at 65°C for 8 hours. The biphasic mixture was cooled to room temperature, diluted with DCM (100mL), and washed with water (2x50mL).

The organic phase was dried over sodium sulfate and the solvent was removed. Silica column chromatography (1% Acetone in Chloroform) gave the pure product as a dark purple solid. (85mg, 64%). 1H NMR (400 MHz, $CDCl_3$): δ 9.04 (d, J = 4.0 Hz, 2H), 8.72-8.53 (m, 8H), 7.85 (d, J = 8.0 Hz, 4H), 7.54 (d, J = 4.1 Hz, 2H), 7.43 (d, J = 8.0 Hz, 4H), 5.23-5.10 (m, 2H), 4.15-4.05 (m, 4H), 3.18-3.10 (m, 2H), 2.29-2.21 (m, 4H), 2.05-1.97 (m, 4H), 1.92-1.87 (m, 4H), 1.81-1.76 (m, 4H), 1.45-1.10 (m, 64H), 0.88-0.81 (m, 18H). MALDI-TOF (*m/z*) M^+ = 1928.813 (Calcd for $C_{124}H_{132}N_6O_{10}S_2$: 1928.944)

Synthesis of 3,6-bis(thiophene-2-yl)-2,5-dihydropyrrolo[3,4-c]pyrrole-1,4-dione, Bare DPP (SI-3). Sodium tert-butoxide (4.3g, 45 mmol) was dissolved in 100mL 2-methyl-2-butanol in a 250mL two necked round bottom flask equipped with a reflux condenser and heated to 120°C. Thiophene carbonitrile (5g, 45 mmol) was added forming a cloudy suspension. Diethylsuccinate (4g, 22.5 mmol) was added dropwise to the mixture over 30 minutes resulting in a deep red solution which was heated at 120°C for an additional two hours. The solution was cooled and the product precipitated by pouring into 420mL 20:1 methanol/12M HCl. The dark red precipitate was collected by filtration and dried to give bare DPP (2.35g, 34%) which was used in the next step without further purification.

Synthesis of 2,5-Bis(1-decyl)-3,6-bis(thiophene-2-yl)-2,5-dihydropyrrolo[3,4-c]pyrrole-1,4-dione, Decyl DPP (SI-4). In a 100mL round bottom flask, **SI-3** (1g, 3.3mmol) was dissolved in DMF (50mL) with potassium carbonate (1.4g, 10.0mmol) and heated to 120°C for one hour before adding bromodecane (2mL, 8.3mmol). The solution was stirred overnight at 120°C, cooled to room temperature and diluted with chloroform. The organic layer was washed with water (3x50mL), dried over sodium sulfate and the solvent was removed under reduced pressure. Silica column chromatography ($CHCl_3$) gave pure product as a purple solid (1.08g, 85%). ^1H

NMR (500 MHz, $CDCl_3$) δ 8.89 – 8.79 (dd, *J* = 4.0, 1.1 Hz, 2H), 7.62 – 7.50 (dd, *J* = 5.0, 1.1 Hz, 2H), 7.23 – 7.20 (dd, *J* = 5.0, 3.9 Hz, 2H), 4.04 – 3.96 (m, 4H), 1.73 – 1.63 (p, *J* = 7.7 Hz, 4H), 1.39 – 1.30 (m, 4H), 1.30 – 1.14 (m, 24H), 0.83 – 0.78 (t, *J* = 7.0 Hz, 6H). MALDI-TOF (*m/z*) M^+ = 580.426 (Calcd for $C_{34}H_{48}N_2O_2S_2$: 580.315)

Synthesis of 2,5-Bis(1-decyl)-3,6-bis(5-bromo-thiophene-2-yl)-2,5-dihydropyrrolo[3,4-c]pyrrole-1,4-dione, Dibromo DPP (SI-5). In a 100mL round bottom flask, **SI-4** (350mg, 0.6mmol) was dissolved in DCM (20mL) and covered with aluminum foil. N-bromosuccinimide (225mg, 1.3mmol) was added in a single batch and the solution was stirred for 48 hours after which a dark purple sludge was formed. The mixture was diluted with MeOH (100mL) and stirred for an additional 2 hours resulting in dark purple needles. The crystallization was finished in the freezer over 1 hour and the product was filtered and washed with hot MeOH to give the product as dark purple fibers (259mg, 59%). Spectroscopic data matched what is given in the literature.[2]

Synthesis of 2,5-Bis(1-decyl)-3,6-bis(5-phenylthiophene-2-yl)-2,5-dihydropyrrolo[3,4-c]pyrrole-1,4-dione, DPP (3). 20mg(0.027 mmol) dibromo-DPP was combined with 40mg(0.33mmol) phenylboronic acid and 90mg(0.65mmol) potassium carbonate in a 2-necked 25mL round bottom flask fitted with a reflux condenser. To the flask was added 5mL toluene, 0.5mL absolute ethanol, and 0.5mL deionized water. The resulting mixture was bubbled with nitrogen for 30 minutes and 5mg(0.004mmol) of $Pd(PPh_3)_4$ was added. The reaction mixture was bubbled with nitrogen for an additional 15 minutes and heated at 65°C for 8 hours. The biphasic mixture was cooled and diluted with 50mL DCM and extracted with water (2x50mL). The organic phase was dried over sodium sulfate and the solvent was removed. Silica column chromatography (DCM/Hexanes 1:1) gave the pure product as a dark purple solid (19mg, 95%).

1H NMR (400 MHz, $CDCl_3$): δ 8.96 (d, J = 4.2 Hz, 2H), 7.68 (d, J = 7.2 Hz, 4H), 7.47 (d, J = 4.1 Hz, 2H), 7.44 (t, J = 7.4 Hz, 4H), 7.37 (d, J = 7.2 Hz, 2H), 4.08 (m, 4H), 1.94 (m, 2H), 1.34 (m, 16H), 0.93 (t, J = 7.4 Hz, 6H), 0.87 (t, J = 6.7 Hz, 6H). MALDI-TOF (*m/z*) M^+ = 732.272 (Calcd for $C_{46}H_{56}N_2O_2S_2$: 732.378)

Spectroscopy of Model Compounds

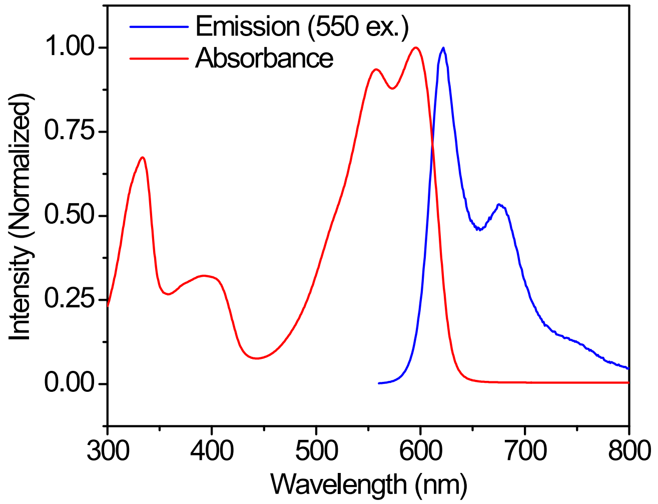

Figure S1: Steady state absorption and fluorescence spectra of **2** in toluene solution. The fluorescence spectrum was measured with an excitation wavelength of 550 nm.

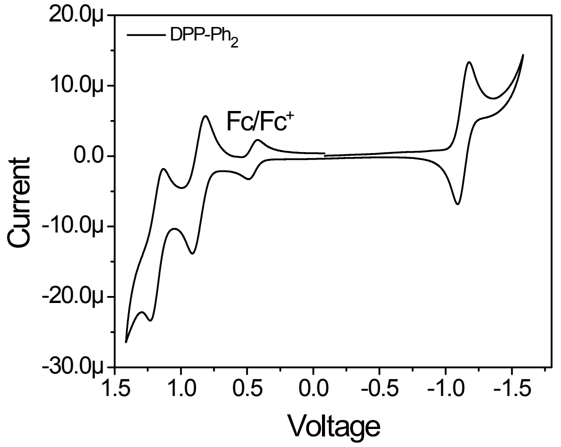

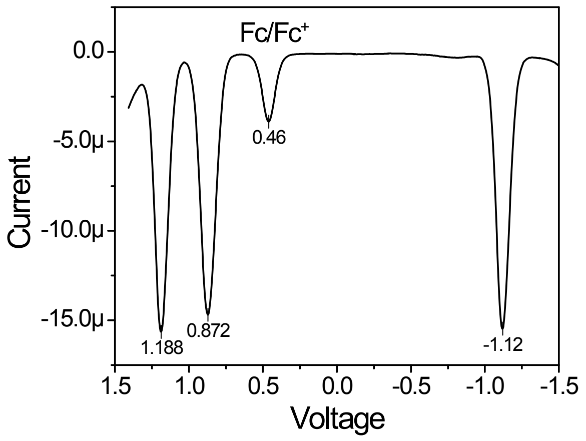

Figure S2: Cyclic voltammetry (left) and differential pulse voltammetry (right) of **2** 1mM in CH_2Cl_2 with 100mM $TBAPF_6$ electrolyte. Spectra are reported vs SCE and were calibrated using Fc/Fc+ as an internal standard.

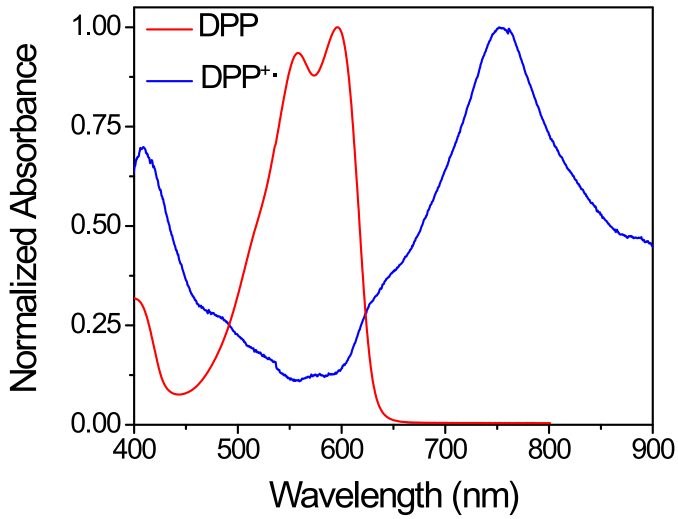

Figure S3: Spectroelectrochemically determined cation absorption of **2** in CH_2Cl_2 compared to ground state absorbance of **2** in CH_2Cl_2. The cation spectrum was generated at +0.9 V vs AgQRE, where bleaching of the ground state was first observed..

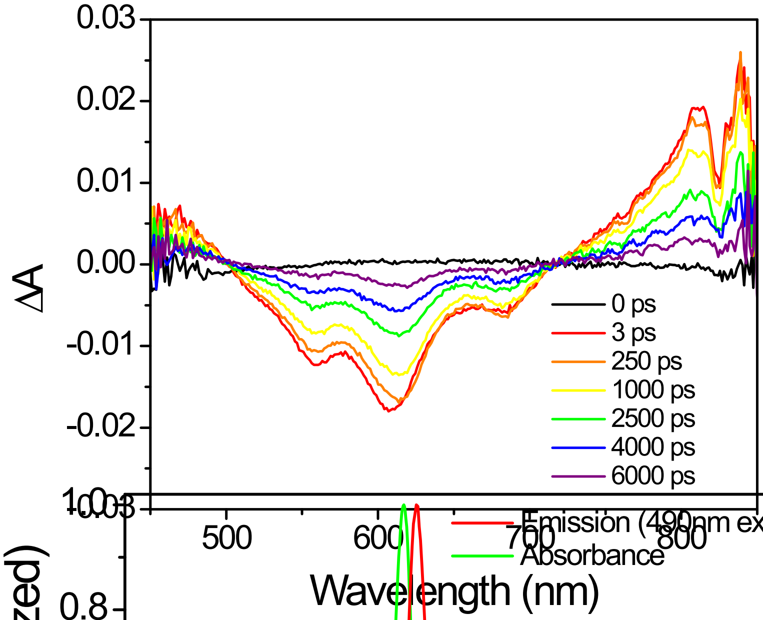

Figure S4: fsTA spectra of **2** in toluene excited at 532nm. The negative features at 550nm and 600nm correspond to ground state bleaching, the feature at 685nm corresponds to stimulated emission and the positive feature at 800nm corresponds to excited state absorption.

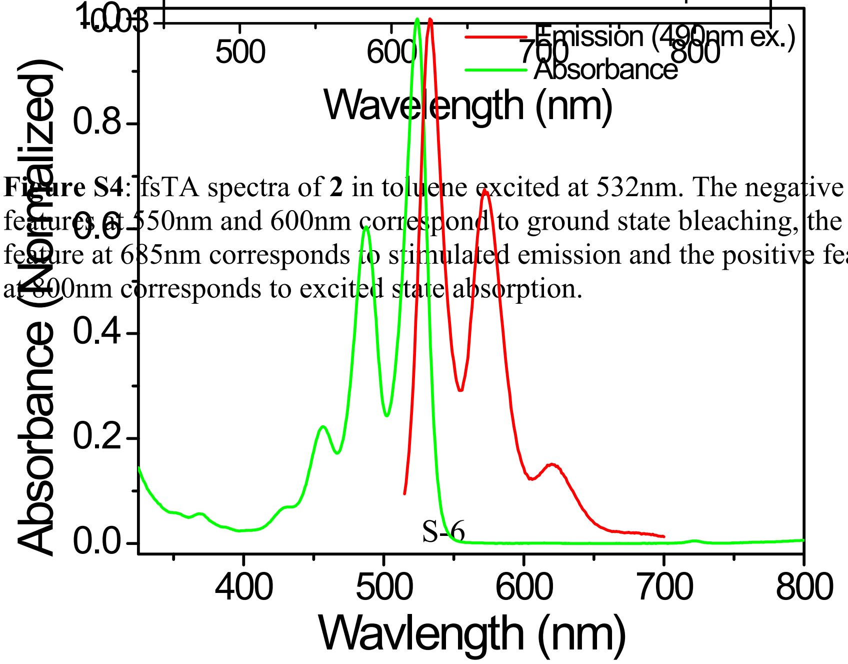

Figure S5: Steady state absorption and fluorescence spectra of **3** in toluene solution. The fluorescence spectrum was measured with an excitation wavelength of 490 nm.

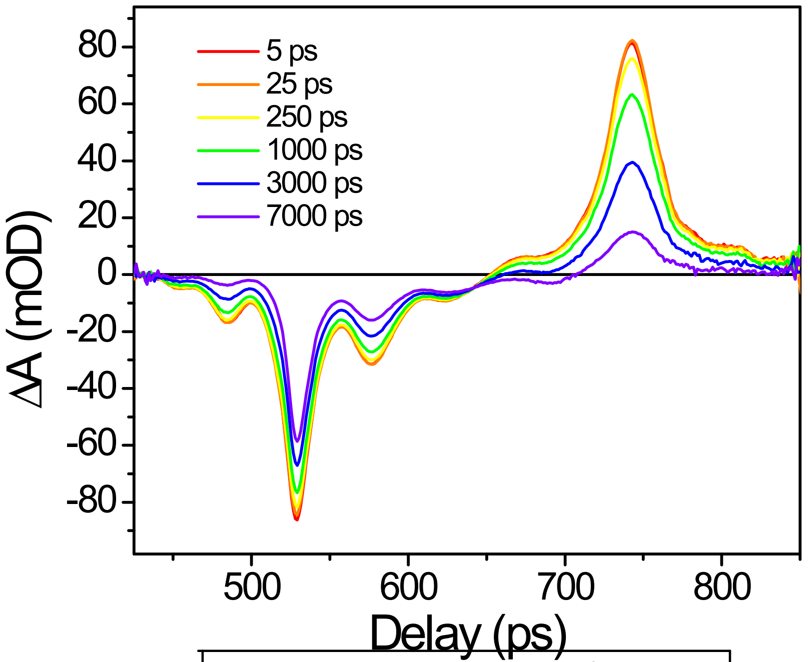

Figure S6: fsTA spectra of **3** in toluene excited at 532 nm. The negative features at 490 nm and 525 nm are ground state bleaching, the features at 585 nm and 615 nm are stimulated emission and the positive feature at 750 nm is excited state absorption.

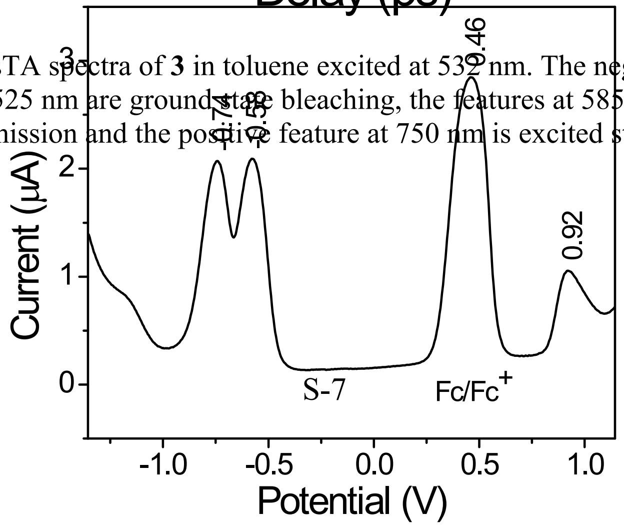

Figure S7: Differential pulse voltammetry of **1,** 0.1 mM in $CHCl_3$ with 100 mM $TBAPF_6$ supporting electrolyte. Voltammograms are reported vs SCE and were calibrated using Fc/Fc+ as an internal standard. Chloroform was used as a solvent because of the low solubility of **1** in CH_2Cl_2. A clean cyclic voltammogram could not be obtained because of the lower solubility.

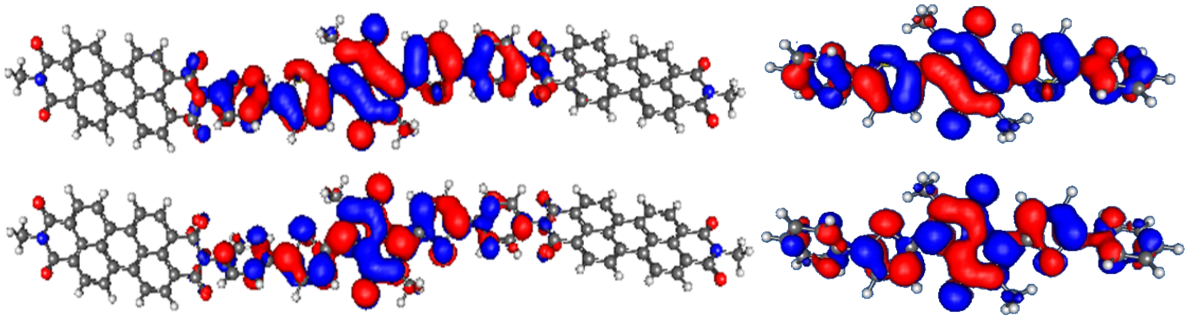

Figure S8: HOMO and HOMO-1 orbitals of **1** triplet (left) and **2** triplet (right) calculated using B3LYP/6-31+G*. The calculated triplet energy of **1** is 0.99 eV and the calculated triplet energy of **2** is 1.02 eV.

REFERENCES:

(1) Wu, Y.-L.; Brown, K. E.; Wasielewski, M. R. *J. Am. Chem. Soc.* **2013**, *135*, 13322.

(2) Kirkus, M.; Wang, L.; Mothy, S.; Beljonne, D.; Cornil, J.; Janssen, R. A. J.; Meskers, S. C. J. *J. Phys. Chem. A* **2012**, *116*, 7927.
